# The dynamics of actin network turnover is self-organized by a growth-depletion feedback

**DOI:** 10.1038/s41598-020-62942-8

**Published:** 2020-04-10

**Authors:** P. Bleicher, A. Sciortino, A. R. Bausch

**Affiliations:** Lehrstuhl für Biophysik E27, Physik-Department, Technische Universität München, Garching, Germany

**Keywords:** Biological physics, Cytoskeletal proteins

## Abstract

The dynamics of actin networks is modulated by a machinery consisting of actin binding proteins that control the turnover of filaments in space and time. To study this complex orchestration, *in vitro* reconstitution approaches strive to project actin dynamics in ideal, minimal systems. To this extent we reconstitute a self-supplying, dense network of globally treadmilling filaments. In this system we analyze growth and intrinsic turnover by means of FRAP measurements and thereby demonstrate how the depletion of monomers and actin binding partners modulate the dynamics in active actin networks. The described effects occur only in dense networks, as single filament dynamics are unable to produce depletion effects to this extent. Furthermore, we demonstrate a synergistic relationship between the nucleators formin and Arp2/3 when branched networks and formin-induced networks are colocalized. As a result, the formin-enhanced filament turnover depletes cofilin at the surface and thus protects the dense, Arp2/3 polymerized network from debranching. Ultimately, these results may be key for understanding the maintenance of the two contradicting requirements of network stability and dynamics in cells.

## Introduction

The polarity of actin filaments is one of the hallmark features of the cytoskeleton, promoting cell dynamics by locally balancing the assembly and disassembly rates of filaments^1,2^. This dynamic turnover of the filaments can^3^, if coordinated by a diverse set of actin binding proteins influence the network architecture, flows and forces within cytosolic fluids^4–6^.

Among the proteins that are involved in these processes, cofilin is a key regulator of actin dynamics that binds and stochastically severs actin filaments, thus creating new filament ends^7,8^. While contributing to the polymerization dynamics by providing additional barbed ends^9^, cofilin simultaneously increases the fraction of monomers in a treadmilling network^10^. This effect is attributed to increased disassembly rates at both ends of cofilin decorated actin in contrast to undecorated actin^11,12^. Decoration occurs preferentially from the pointed ends, since cofilin has a higher affinity towards ADP-actin^9^. Although cofilin bound monomers have a decreased nucleotide exchange rate^12^, the actin binding protein CAP1 acts as a nucleotide exchange factor and thus replenishes the active monomer pool in the prescence of ATP^13,14^ alongside profilin^15–17^. Additionally, CAP1 increases the overall disassembling activity of cofilin^18^.

Formin is a processive nucleator and elongator protein that is able to polymerize actin in a force-sensitive manner^19^. It has previously been shown that single actin filaments can be rapidly polymerized by formin and simultaneously be severed and disassembled by cofilin^20,21^. In combination with capping protein and cofilin, skyrocketing elongation rates due to funneling of monomers have been reported^10^. While these processes have been identified by the *in vitro* reconstitution of single filament dynamics, how these effects can be scaled up to the mechanisms governing the dynamics of actin networks is so far unknown^22^. Recent publications emphasize the importance of such systems, as certain effects can only be achieved as a consquence of global actin turnover^23–25^. Here, the competition for a shared actin pool, as well as maintained gradients of the actin monomer concentration are of significance, which are not taken into account from a single filament perspective. Even less is known about how the dynamics of different network types interact with each other. Despite the importance of the interplay and competition between different nucleation machineries, how the growth and turnover dynamics of actin would be affected remains elusive^26^.

Here we show that localizing the nucleation and elongation of actin filaments onto a bead surface using the actin elongator formin enables us to reconstitute active turnover of polarized high density actin networks and to identify effects that result from the local depletion of monomers. These effects occur only in high density networks, where the elongation of filaments is restricted to the functionalized surface and affected by the diffusion limit, which dominates the observed dynamics. We find that the constant exchange of monomers from the pointed end to the barbed end depends strongly on the effective local cofilin concentration. A high turnover rate is observed only at intermediate cofilin concentrations. By locally combining different nucleators and thus network architectures, we show how globally treadmilling filaments protect branched networks from disassembly in a synergistic manner. When branched networks polymerized by Arp2/3 are present alone, they are rapidly debranched already at nanomolar cofilin concentrations^27,28^. However, by combining formin and Arp2/3 to yield two different network architectures, branched networks remain unaffected by the disassembly activity of cofilin at cofilin concentrations up to 2 µM. This effective protection is likely due to the localized depletion of cofilin, induced by a constant binding to the simultaenously present dynamic actin network and leading to an intrinsic turnover maintained by formin. Together, this work demonstrates that the dynamics of actin networks are sensitive to localized collective interactions of Arp2/3, formin and cofilin, thus extending our understanding of these interactions from a single molecule up to a whole network level.

## Results

**Figure 1:**
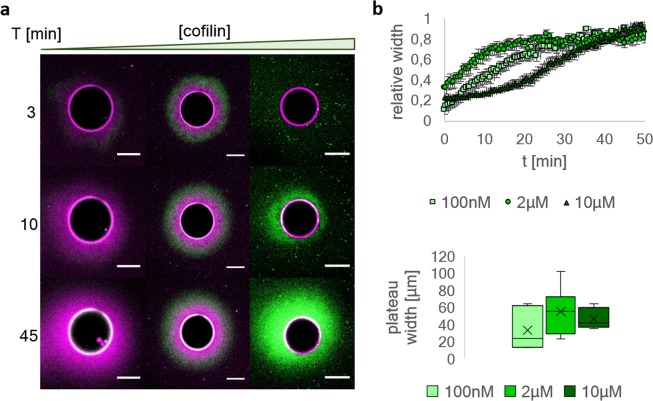
By varying the cofilin concentration networks can be divided into three regimes. (**a**) Network polymerization on formin functionalized spheres in the presence of 12 µM profilin, 100 nM capping protein, 1 µM CAP1, 5 µM actin and 100 nM cofilin (left column), 2 µM cofilin (middle column) and 10 µM cofilin (right column), actin is shown in magenta, cofilin is shown in green. At 100 nM cofilin (left column), the network consists mostly of cofilin undecorated actin. At 2 µM (middle column), the cofilin decorated and undecorated regions of the networks are balanced and at 10 µM cofilin (right column) the network is mostly decorated. (**b**) The growth kinetics of all networks are affected by the cofilin concentration. Here, the network width is plotted against time. At 2 µM cofilin networks reach their plateau width fastest. This can be explained by the increased net turnover compared to the 100 nM cofilin network. At 10 µM cofilin, the net growth rate of the cortex is slowed down by the excess of cofilin and the resulting enhancement of disassembly rates. The absolute values of plateau widths (n = 6) vary at all concentrations and changes depending on the cofilin concentration are within the measurement error. The first data points at t = 0 min represent the first frame of the measurement and were taken exactly 3 min after initiation of the experiment in all cases. All scale bars represent 50 µm.

Polarized actin networks were polymerized from NiNTA agarose beads with diameters of 60 µm on average, which were functionalized with formin (6xHis-mDia1)^29,30^. Formin has been reported to stick to the barbed ends of filaments and elongate them processively by recruitment of actin monomers^29^. To prevent background polymerization, we added profilin and capping protein to the solution (12 µM profilin, 100 nM capping protein and 5 µM actin with 10% fluorescently labeled monomers) which binds to actin monomers and thereby suppresses spontaneous nucleation. Additionally, profilin-actin increases the efficiency of formin by bringing actin monomer to the filament barbed end^31^. By these means we can spatially control nucleation and elongation of the actin filaments. We observe that actin is nucleated and elongated from the bead surface only. In the absence of cofilin, phalloidin staining is possible, revealing that filaments are unbundled and that the barbed ends are attached to the formins at the surface while the pointed ends extend normal to the bead surfaces into the solution (Fig. 1a, Supplementary Fig. S1). Addition of monomers labeled with a different fluorescent color shows that monomers are built into the network from the functionalized surface (Supplementary Fig. S2). This confirms that the barbed ends are indeed located at the bead surface, thus confirming the filament orientation.

Addition of cofilin results in the disassembly of the filaments in a concentration dependent manner^32–34^. Once depolymerized by cofilin, ADP monomers are recharged with ATP by the presence of CAP1 (1 µM). In their ATP state the monomers are able to rebind profilin, hence forming a continuous polymerization/depolymerization turnover cycle of the actin network (Supplementary Fig. S4a). To confirm the functionality of this system, the resulting dynamics are quantified by means of time resolved confocal microscopy and by using a fluorescent mCherry-cofilin construct (Supplementary Fig. S3). At a concentration of 2 µM, cofilin (Fig. 1a middle column, Supplementary Fig. S4b) decorates the aged part of the filaments but is found only sparsely close to the surface of the beads. This indicates that nearby the surface, the filament ends contain only freshly polymerized ATP- or ADP-Pi-actin. This is observed over an extended time period of 1 h, much longer than the typically observed time necessary for ATP hydrolyzation in filamentous actin^35^ (Movie S1). Furthermore, at 2 µM cofilin propulsion and spinning of the beads can be observed on a timescale that outlives a single polymerization cycle (Supplementary Fig. S5, Movie S2).

**Figure 2:**
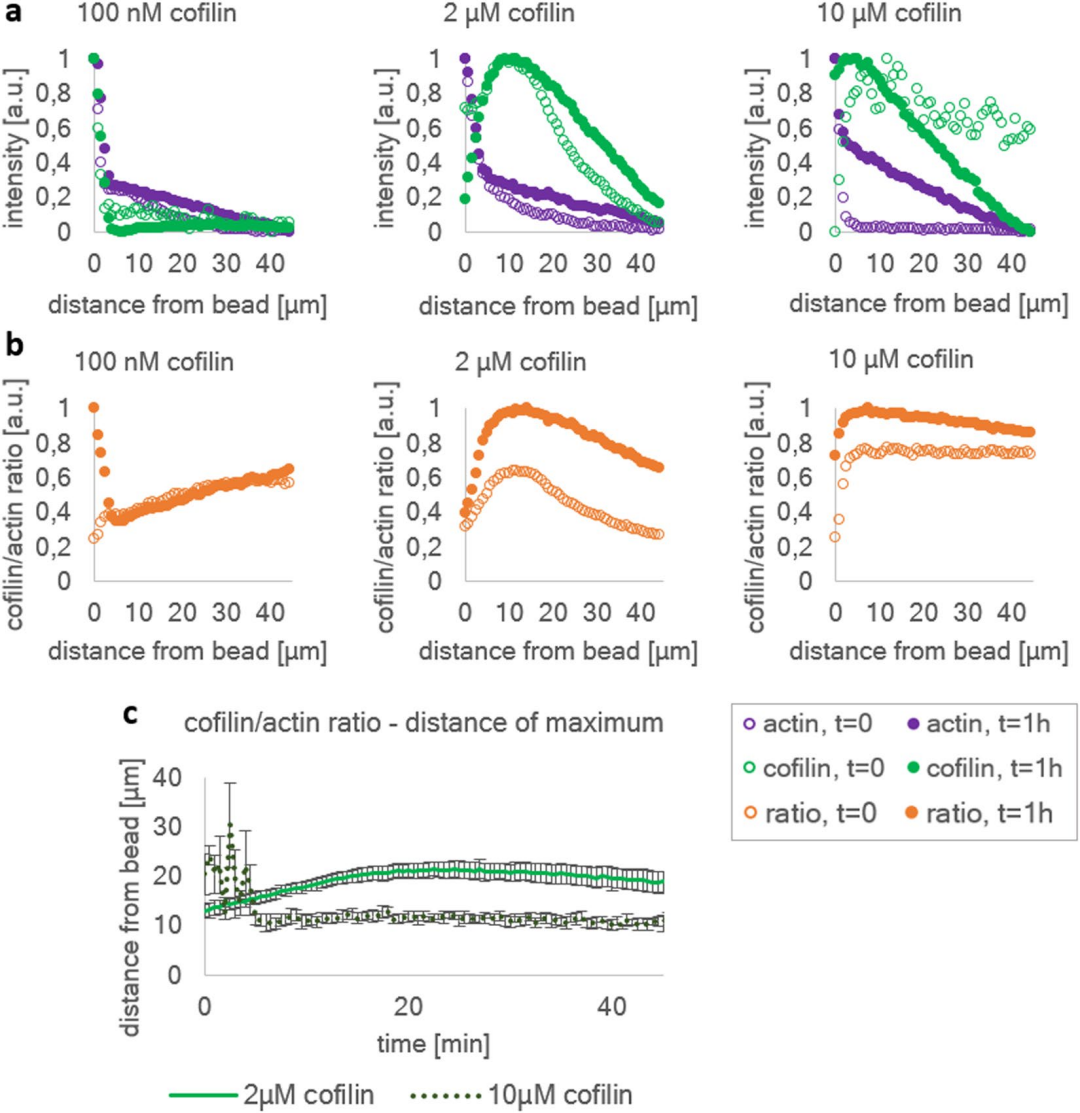
Radial intensity profiles of actin networks polymerized on agarose beads in the presence of different cofilin concentrations. (**a**) Actin intensity profiles (magenta) display a similar trend in shape and width at all cofilin concentration. The cofilin intensity profiles (green) depend however strongly on the used cofilin concentration. At 100 nM cofilin, the cofilin profile is low and flat throughout the cortex, however, after 1 h of polymerization an increase towards the outside solution where the pointed ends are located can be observed. This indicates that most of the actin in this network is not decorated with the exception of the pointed ends at around 40 µm from the bead surface where the network ends. At 2 µM and 10 µM cofilin a maximum in the cofilin intensity profile is visible. Only at 2 µM cofilin, a zone exists in front of the maximum where cofilin is locally sparse. (**b**) Comparison of the cofilin to actin intensity ratios. These ratios reflect the fraction of cofilin decorated actin of the network, thus identifying regions in the network that are most affected by the effects of cofilin. At 2 µM and 10 µM cofilin a decoration maximum is visible at a distinct distance of 15 µm from the surface for 2 µM cofilin and 10 µm from the surface for 10 µM cofilin. At 100 nM cofilin no maximum can be fitted to the ratios, but the profiles display a trend to reach a plateau at around 40 µm where the networks end. (**c**) Fitting maxima to the cofilin/actin ratios for multiple beads (n > 3) visualizes the distance towards the bead surface of the maximum over time. The first data points at t = 0 min represent the first frame of the measurement and were taken exactly 3 min after initiation of the experiment.

By varying the cofilin concentration, we are able to tune the ratio between cofilin decorated and undecorated actin in the formed networks. We divide the effect of cofilin into three concentration regimes (Fig. 1). At 100 nM cofilin the network is mostly undecorated by cofilin, at 2 µM the length of decorated and undecorated actin is more balanced and at 10 µM most actin is in the cofilin-decorated state (Figs. 1a and 2a). In Fig. 1b we show that there is no significant difference between the absolute values of the network widths at different cofilin concentrations. This can be explained by the fact that labeled monomers are employed to determine the network width. Labeled monomers do not allow to distinguish between actin filaments and severed actin fragments. Thus, the determined values do not take into account what fraction of the network has been severed. The network width has been determined by defining a threshold above the average background fluorescence intensity. Remarkably, in the intermediate regime (2 µM cofilin) the plateau width of the network is reached about 10 min faster than in the other two regimes. Hence, the necessary time for the network to reach its plateau increases when the concentration deviates from the intermediate regime of 2 µM. The slowdown at high concentrations can be partially explained with oversaturation of filaments with cofilin, as the increase of the net polymerization rate by severing only occurs to the point of saturation of the filament^12^. When oversaturated with cofilin, cofilin can also bind to the ATP-decorated part of the filament, thus the sites between the ATP and ADP-Pi part of the filament where severing preferentially occurs is not present. However, due to the activity of CAP1, the disassembly of saturated filaments is promoted^14^. Therefore, a biphasic contribution of cofilin to the polymerization rate due to nucleation by severing is not expected, as the decorated parts of the filaments are prone to be disassembled by the interactions of cofilin and CAP1. Consequently, the growth rate of the network width should only depend on the balance of the elongation rate at the surface and the depolymerization rate at the pointed ends.

The radial intensity profiles of actin and cofilin demonstrate the development of the network width and composition over time (Fig. 2a). The actin intensity profiles (magenta) are measured by means of covalently labeled monomers and therefore represent the local density of the network. All networks are densest at the surface and, as a consequence of the spherical bead geometry, less dense with increasing distance. Over time, in all regimes networks grow denser. An intensity maximum can be observed directly on the surface of the bead, most likely due to the density of nucleators that could be saturated by monomers. Since cofilin is not expected to affect the binding of actin to formin, this intensity value is the same in all cofilin concentrations and is used to normalize the data. The most notable difference between the initial and equilibrated state can be observed at 10 µM cofilin. In accordance to our previous observations, there is almost no actin polymerized in the initial state and the net elongation is slowed down by the excess of cofilin. After equilibration however, the actin profiles in all regimes are similar in relative intensity, width and slope.

The cofilin profile (green) is relatively low and flat in the 100 nM regime, indicating that most actin in the network is undecorated. Cofilin also binds to the actin at the bead surface, since the net growth kinetics are slowed down compared to intermediate cofilin concentration as shown before (Fig. 1b). Slower elongation leads to a higher concentration of ADP-actin closer to the bead surface. This results in a peak in the cofilin intensity at the bead surface, where the actin concentration is highest. At 2 µM cofilin, the cofilin profile has a peak at 12 µm from the bead surface that grows wider during equilibration while the position of the maximum does not change. In front of this maximum an area can be identified where cofilin is sparse. At 10 µM cofilin a similar peak is visible even closer to the bead, which exhibits a similar broadening, however in this regime the apex migrates closer towards the surface over time. Therefore, a zone where cofilin is sparse disappears. The broadening of the profiles can be explained by the activity of cofilin. By severing and disassembly of fragments, cofilin creates a fraction of elements that are diffusive, unlike the network. Until the severed cofilin decorated actin fragments are completely disassembled, they can diffuse freely and therefore likely contribute to cofilin peak broadening over the course of the experiment.

The ratio of cofilin to actin intensities (Fig. 2b) can be used as relative measure to estimate the fraction of decorated to undecorated actin, which can be assumed to be proportional to the severing and disassembling activity of cofilin due to the presence and activity of CAP1. CAP1 not only acts as a nucleotide exchange factor, but also promotes the disassembly of cofilin decorated actin filaments^14,36,37^. Because of the affinity of cofilin towards ADP-actin, the ratio profiles represent the distribution of the transition probability from ATP-monomers to ADP-Pi and ADP-monomers, of which the latter is more likely to be decorated by cofilin. Since severing occurs preferentially at the boundaries between bare and decorated actin^9,11,38^, we can pinpoint areas where severing of filaments and the resulting filament turnover is highest.

At 100 nM cofilin, the ratio is initially lowest close to the bead and increases until a plateau is reached at 40 µm distance. In contrast, at 2 µM and 10 µM cofilin the profiles display a peak in the vicinity of the surface and decrease from there. We fit maxima for 2 µM and 10 µM cofilin (Fig. 2c) to the decoration profiles of multiple beads and find that over time all regimes have a tendency towards a decoration maximum at a distinct distance, depending on the cofilin concentration: 15 µm at 2 µM cofilin and 10 µm at 10 µM cofilin. For 100 nM cofilin no clear boundary within the network was detectable and 40 µm was determined to be the total width of the actin network. Because of the constant elongation of filaments, it is expected that the distance of the boundary increases when the net turnover is higher, which suggests the intermediate cofilin concentrations of 2 µM to be the internally most dynamic regime.

At all cofilin concentrations an equilibrium is reached after 20 min (2 µM cofilin), 30 min (100 nM cofilin) and 50 minutes (10 µM cofilin) where neither the length of the network, nor the ratio between width of cofilin decorated actin and actin changes. This suggests that a steady state of collectively treadmilling filaments is reached. To confirm that this equilibrium is in fact a dynamic steady state, we use FRAP measurements.

**Figure 3:**
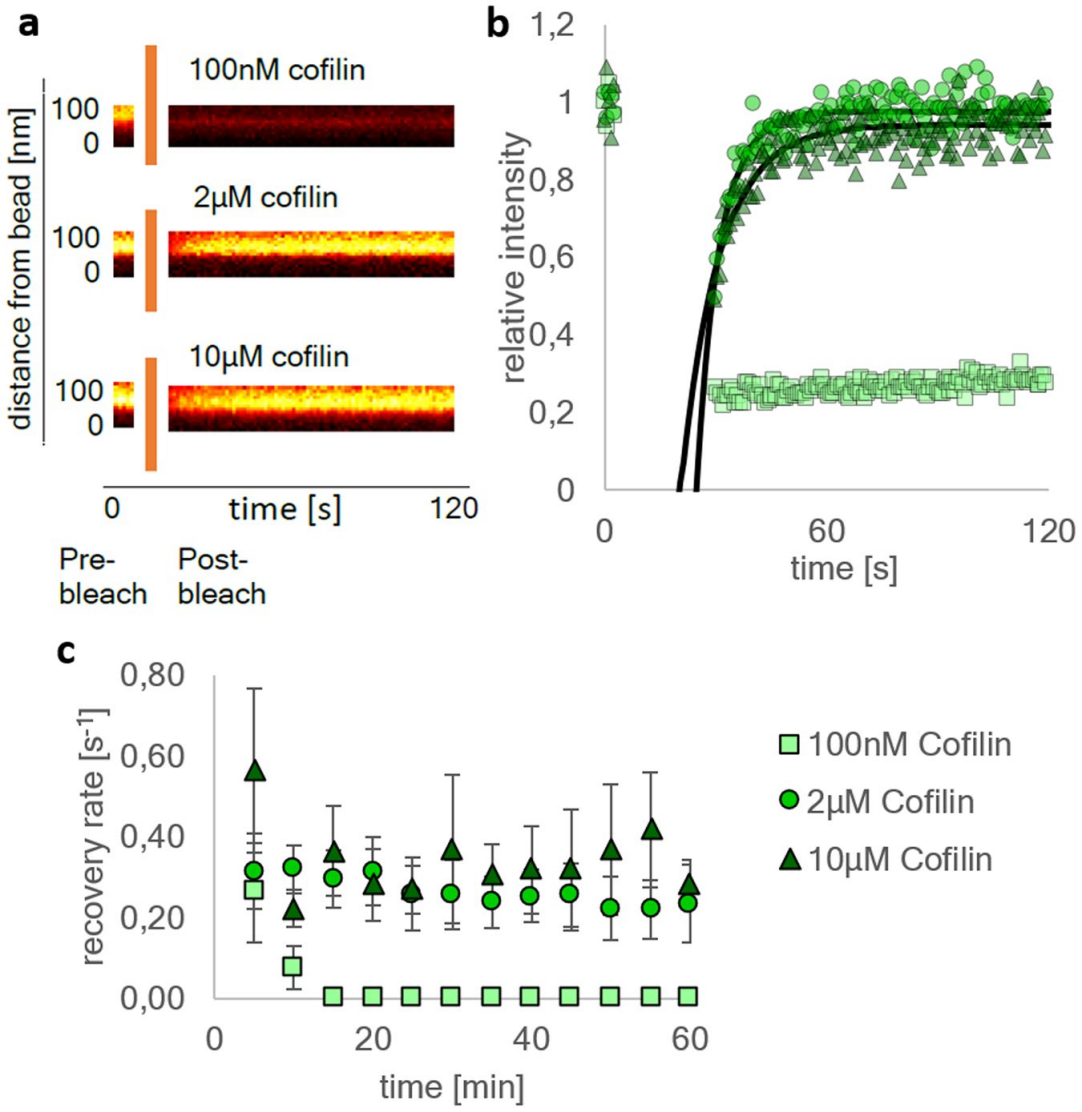
FRAP measurements of Atto 488 NHS-ester labeled actin networks at different cofilin concentrations. A spot directly on the bead surface has been bleached. (**a**) A kymograph of the bleached spot depicts the intensity profile over two minutes. Shown are the frames pre-bleach and post-bleach. At 2 µM cofilin and 10 µM cofilin the recovery is faster compared to the 100 nM network. Additionally, the kymographs show that the recovery emerges from the bead surface (bottom) towards the exterior solution (top). The measurements for the kymographs were started 12 minutes after the initiation of the experiments (**b**) The fluorescence intensity corresponding to the bleached spots in the kymographs is plotted against time. There is no significant difference visible between the networks at 2 µM cofilin (circles) and 10 µM cofilin (triangles). A single exponential fit (black lines) is added to both concentrations. However, at 100 nM cofilin (squares) the recovery is slowed down to the point where a single exponential cannot be fitted to the curve. This suggests that the fluorescence recovery is strongly affected by the disassembly and replenishment activity of the cofilin machinery. (**c**) By fitting a single exponential where it was possible, recovery rates can be extracted. Another example for an exponential fit is shown in Supplementary Fig. S7. The extracted fluorescence recovery rates are plotted over time for multiple beads. The recovery rates at 2 µM and 10 µM cofilin display the same trend over time, while the recovery rates at 100 nM cofilin decrease after 10 minutes of polymerization. After ten minutes, the recovery was so slow that an exponential cannot be fitted anymore and the recovery rate was assumed to be zero. The networks at 2 µM and 10 µM cofilin do not show this decrease. The first data points at t = 0 min represent the first frame of the measurement and were taken exactly 3 min after initiation of the experiment in all cases.

To this end a region directly at the bead surface is bleached and the recovery of the fluorescence intensity measured at different time points (Fig. 3a–c). At 100 nM cofilin, the recovery time is slower than 0.1 s^−1^ as soon as 10 minutes after the beginning of the experiment, which can be explained by monomer depletion. At 2 µM and 10 µM cofilin, these rates remain constant above 0.2 s^−1^. Hence, disassembly and the recovery of the monomer pool can be accounted for maintaining the fluorescence recovery in these networks. Additionally, we performed FRAP experiments in the absence or presence of CAP1 (Supplementary Fig. S7). For all cofilin concentrations the recovery rates are significantly faster when CAP1 is present, revealing the contribution of the nucleotide exchange factor. Hence, all regimes display dynamics at the bead surface that strongly depend on the presence of cofilin and CAP1.

To address the question how the dynamics of different network types interact with each other, we test the effect of cofilin on actin networks polymerized by Arp2/3. To this end we functionalize beads with VCA (10 nmol per ml bead resin) which activates the Arp2/3 complex (300 nM) and thus initiates the formation of a localized branched actin network at the bead’s surfaces. Already nanomolar concentrations of cofilin suffice to debranch Arp2/3 bound filaments rapidly^39^.

**Figure 4:**
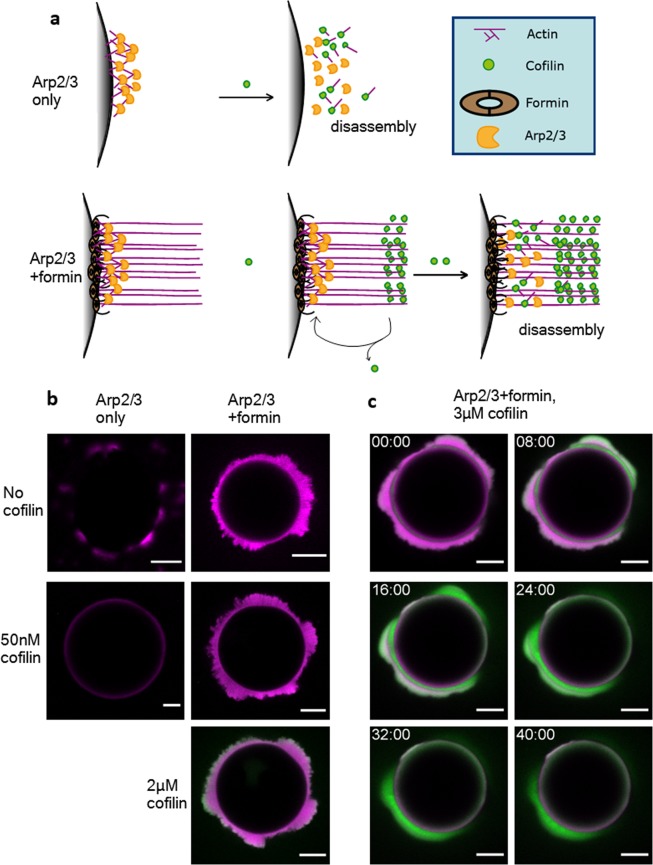
Colocalization of branched Arp2/3 polymerized networks with globally treadmilling formin networks. (**a**) Proposed model for the disassembly of branched networks in the absence or presence of formin. Arp2/3 (orange) is activated by His-VCA, which is immobilized on the NiNTA functionalized bead surface (10 nmol per mL of bead resin) and a branched actin (magenta) network forms. When cofilin (green) is present, the branches are disassembled. Therefore, no network is polymerized already in the presence of nanomolar cofilin concentrations at the employed nucleator density. When formin (brown) is also present (5 nmol per mL of bead resin each), a colocalized network forms. By binding to the continuously polymerizing formin network, a steady state is achieved that locally depletes cofilin. Therefore, the branched network is still intact at concentrations up to 2 µM cofilin. Disassembly of the Arp2/3 polymerized network occurs only at higher concentrations. The illustrations are designed using Adobe Illustrator CS3 graphics creation software retrieved from https://adobe.com/products/illustrator. (**b**) Beads are functionalized with His-VCA with or without His-formin simultaneously. At 50 nM cofilin, no branches form when VCA and Arp2/3 are present alone, however, when formin is colocalized, a dense branched cortex forms that reaches a width of 5–10 µm. The density of the branched network is 6 times higher, hence a present formin network appears too dark to be resolved simultaneously (Supplementary Fig. S8). The branched network remains stable in the presence of the formin network up to concentrations of 2 µM cofilin. Here, the tips of the branches that point away from the bead start to be decorated by cofilin. All beads were incubated in the corresponding cofilin concentration until there was no visible change to the network width or cofilin decoration for at least 10 minutes. (**c**) By increasing the cofilin (green) concentration above 2 µM, the actin (magenta) ends of the network that point towards the bead surface are also decorated. Under these conditions, the branched network is gradually disassembled. In all images, actin is shown in magenta, cofilin is shown in green. The first data points at t = 0 min represent the first frame of the measurement and were taken exactly 3 min after initiation of the experiment in all cases. All scale bars represent 20 µm, the depicted timescale is in minutes.

Indeed, no network can be polymerized in the presence of 50 nM cofilin (Fig. 4b, left column). Yet, when both Arp2/3 and formin are present and mixed on the same bead surface (5 nmol each per mL bead resin), both network types polymerize simultaneously and colocalize. The formin-induced networks exhibit dynamic turnover behavior, reaching network widths of 50 µm while the Arp2/3 induced branched network continues to polymerize reaching only widths of 5 to 10 µm but with a much higher filament density (6x higher density in branched compared to parallel network, Supplementary Fig. S8). By gradually increasing the concentration of cofilin, we find that the branched network stays unaffected by the activity of cofilin even up to cofilin concentrations of 2 µM (Fig. 4b, right column). Only once the cofilin concentration is increased from 2 µM to 3 µM does the branched network disassemble completely (Fig. 4c, Movie S3). Thus, formin induced actin networks which extend beyond the Arp2/3 formed network shield the Arp2/3 networks against the effect of cofilin. Dynamic formin networks alone create a zone close to the surface where cofilin is sparse. This effect is most pronounced at 2 µM cofilin, however, at higher concentration this zone is getting smaller (Fig. 2a,c). In networks with formin and Arp2/3 complex colocalized, the cofilin exclusion zone is also present (Fig. 4b). Thus, it enables Arp2/3 networks to remain stable against debranching by cofilin even at high cofilin concentrations through the control of the local free cofilin concentration by the competing network (Fig. 4a).

## Discussion

The presented experiments show that formin induced polarized network growth can reach the highest recovery rates of up to 0.8 s^−1^ at concentrations above 2 µM cofilin (Fig. 3). Compared to single filament dynamics^21,40^, the elongation speeds of networks are 10 times slower (Supplementary Fig. S6) when no monomer recovery is present. This can be attributed to the fact that the polymerization kinetics of networks is diffusion limited and that the actin monomers are locally depleted at the surface of the bead by the presence of a high density of active filament ends. Similar effects were observed for branched networks polymerized by Arp2/3^23,41^.

The diffusion times limiting the growth rate depend critically on the cofilin concentration. This is best seen by analyzing the intensity profiles to identify areas in the network that are decorated by cofilin and identify the distribution of boundaries of bare to decorated actin, where the severing activities are highest. Indeed, we find that the distance at which the ratio of cofilin to actin is highest depends strongly on the cofilin concentration. At 100 nM mostly undecorated actin exists and only the bulk actin monomer concentration can be used for elongation, which needs to diffuse over the total actin cortex thickness of 40 µm. At 2 µM cofilin a clear cofilin decoration maximum is found at approximately 15 µm from the surface. Hence, for the latter the monomer diffusion time would be 7 times shorter than for 100 nM cofilin concentration. The recovery rates in these two equilibrated states drop on average by a factor of about 6 as measured by FRAP, which fits very well the diffusion time difference. The replenishment of the active monomer pool is achieved by the presence cofilin and enhanced by at least a factor of 2 by the addition of CAP1 (Supplementary Fig. S7), achieving enhanced severing and nucleotide exchange. Thus, the increase of local disassembly and recycling of the monomers to ATP monomers at higher cofilin concentrations leads to an increase in the monomer flux and accounts for high turnover rates in even dense networks. Together, the polar networks described here are self-sustained by a constant flow of recycled monomers from the pointed ends to the barbed ends.

Geometrical effects such as the filament twist^42,43^ come to mind to explain the described effects, however the filaments in the presented experiments are not trapped and can therefore twist and fluctuate freely. Therefore, the filament twist induced by formin is unlikely to significantly affect the cofilin binding here. Also, the contribution of the global actin concentration cannot fully explain the difference in turnover rates (Fig. 3), as even under conditions where maximal amounts of monomers are built into a network, no more than 22% of the global actin can be depleted (Supplementary document S9). A steady state mechanism of kinetic binding towards a continuously polymerizing network, linked with local depletion of monomers is most likely, as it explains all the presented effects.

At 2 µM cofilin, the continuous elongation of actin creates a zone between the functionalized surface and the decoration maximum at 15 µm distance, where cofilin is locally sparse, as the local production of actin is faster than the diffusion of unbound cofilin towards the exclusion zone. The intrinsic actin turnover dynamics are not affected by increasing the cofilin concentration from 2 µM to 10 µM (Fig. 3b), however, due to the availability of more cofilin, the zone where cofilin is excluded becomes smaller (Fig. 2a). In sum, at 100 nM cofilin, the turnover dynamics are slow, due to the depletion of both actin monomers and unbound cofilin at the elongation site. At 2 µM cofilin, the turnover dynamics are fast due to disassembly of actin and replenishing of the monomer pool. However, the fast turnover and binding kinetics deplete cofilin locally, thus creating a zone directly at the elongation site where cofilin is sparse. When increasing the cofilin concentration even higher, to 10 µM, the intrinsic turnover dynamics remain fast due to the monomer recycling, but the sparseness of cofilin at the elongation site disappears due to the availability of unbound cofilin.

As fast turnover dynamics occur at significantly higher concentrations of cofilin than those observed for Arp2/3 induced networks, the question arises of how the dynamics of these two types of networks interact. We find that branched networks only remain unaffected by cofilin, even at concentrations up to 2 µM, when polar formin-nucleated networks are simultaneously present and deplete the cofilin locally. Thus, they prevent the diffusion of free cofilin towards the Arp2/3 generated network by binding and the Arp2/3 induced network polymerizes unaffected in the zone where cofilin is depleted. Therefore, the presence of the actin network itself sets the effective local concentration of cofilin by its local depletion by binding. This in turn determines the resulting local effects of cofilin on the network dynamics. Such localized depletion feedback loops may turn out to be essential for explaining the observed localized organization of dynamic actin networks in cells. This work extends the concept of a global treadmilling process, where coexisting networks are modulated by a common actin monomer pool^24,44^ to also include the localized depletion effects of the actin binding partners. Thus it is not only the actin monomer availability which regulates the homeostasis of actin cytoskeletal networks, but the local availability of depolymerization factors and accessory proteins as well^25,45^.

The resulting effects are synergistic in nature and can ultimately be key to our understanding of the observed specificity of maintaining the competing and local demands of stability and dynamics of actin networks in cells.

## Methods

### Protein purification

Actin was purified from rabbit skeletal muscle^46^. No rabbits were directly involved in the study. Monomeric actin was stored at 4 °C in G-Buffer (2 mM Tris, 0.2 mM ATP, 0.2 mM CaCl_2_, 0.2 mM DTT and 0.005% NaN_3_, pH 8.0). For fluorescent actin, monomers were labelled N-terminally with Atto 488 NHS-ester (Jena Bioscience). Formin (mDia1^29,30^) was expressed with an N-terminal His-tag in BL21 Codon Plus DE3-RIPL (Agilent Technologies) by means of auto-induction^47^. Here, an inoculate was incubated in LB medium at 37 °C for 6 h and afterwards added to auto-induction medium: 928 mL ZY (12 g/L tryptone, 24 g/L yeast extract), 1 mL 1 M MgSO_4_, 20 mL 50 × 5052 (0.5% glycerol, 0.05% D-glucose, 0.2% α-lactose) and 50 mL 20x NPS (0.5 M (NH_4_)_2_SO_4_, 1 M KH_2_PO_4_, 1 M Na_2_HPO_4_), supplied with 4 times the usual amount of antibiotics. The BL21 cells were incubated vigorously shaking for 6 h at 37 °C and subsequently incubated for 60 h at 15 °C. Cells were harvested by centrifugation and resuspended in soni-buffer (50 mM Na_2_HPO_4_, 50 mM NaH_2_PO_4_, 300 mM NaCl, pH 8.0), supplied with 20 mM imidazole, 50 µg/mL lysozyme, 100 U/mL Benzonase (Merck) and EDTA-free protease inhibitor cocktail (Roche). The cells were disrupted 2 times in a French press, centrifuged (39800 xg at 4 °C for 1 h) and the filtrated lysate was purified by Ni-NTA affinity chromatography on cOmplete Ni-NTA resin (Roche) in soni-buffer. The buffer was exchanged by passing the purified formin through a NAP-25 size exclusion column (GE Healthcare) equilibrated in PBS (10 mM phosphate buffer, 140 mM NaCl, 3 mM KCl, 2 mM DTT, pH 7.4) and the eluted formin subsequently stored at −80 °C. Cofilin (hCof1) and mCherry-cofilin were expressed as a glutathione S-transferase (GST) fusion protein in a pGEX-6P2 vector. The BL21 cells were incubated at 37 °C in LB medium supplied with 0.5 M NaCl and 2 mM betaine until they reached an OD_600_ of 0.5. The cells were incubated at 25 °C for 1 h and subsequently induced by addition of 0.5 mM IPTG. After expression overnight, cells were harvested and the resulting cell pellet dissolved in TBSE (25 mM Tris, 300 mM NaCl, 1 mM EDTA, 1 mM DTT, pH 7.4) supplied with 50 µg/mL lysozyme, 100 units/mL Benzonase (Merck) and EDTA-free protease inhibitor cocktail (Roche). The cells were disrupted in a French press, centrifuged (39800 × g at 4 °C for 1 h) and the filtrated lysate purified by affinity chromatography via Glutathione Sepharose 4B (GE Healthcare). The protein was eluted by on-column cleavage during incubation with PreScission Protease (GE Healthcare). The eluted cofilin was dialyzed against cofilin-buffer (10 mM Tris, 150 mM NaCl, 2 mM DTT, pH 7.4) overnight and stored at −80 °C. CAP1 was expressed in a pET-28b(+) vector by incubating BL21 in LB medium at 37 °C until the cells reach an OD_600_ of 0.6. The expression was started by induction with 0.5 mM IPTG and carried out for 16 h at 18 °C. The protein was purified as described previously for formin, but in TBSE at pH 8.0 (25 mM Tris, 300 mM NaCl, 1 mM EDTA, 1 mM DTT, pH 8.0). After Ni-NTA affinity chromatography, the eluted protein was dialyzed against CAP1 buffer (25 mM Tris, 150 mM NaCl, 1 mM DTT, pH 8.0) and thrombin (25 U per mL of eluate) was added to remove the N-terminal His-tag. After incubation for 2 h at 4 °C on a horizontal shaker, Ni-NTA resin (100 µL per mL of eluate) and para-aminobenzamidine resin (GE Healthcare, 50 µL per mL of eluate) was added and incubated for 1 h at 4 °C on a horizontal shaker to remove uncleaved His-CAP1 as well as thrombin. Afterwards, the cleaved CAP1 was collected by passing the eluate and resin through a clean, disposable column. Finally, the eluate was passed through a NAP-25 size exclusion column equilibrated in CAP1 buffer and the collected protein was stored at −80 °C. All other proteins (profilin, capping protein, Arp2/3 and VCA) were purified as described previously^48^.

### Vector construction

To insert the cofilin sequence (hCof1) in a pGEX-6P2 vector, it was amplified by PCR using the Primers 5′- ATATATGGATCCATGGCCTCCGGTGTGGCT-3′ and 5′- GCGCATGAATTCCTACAAAGGCTTGCCCTC-3′ and inserted as a BamHI-EcoRI fragment. The mCherry-cofilin fusion protein in pGEX-6P2 was obtained by amplifying each sequence individually first using the overlapping primers 5′- ATATATGGATCCATGGCCTCCGGTGTGGCT-3′ and 5′- CGGCACCAGGCCGCTGCTCAAAGGCTTGCC-3′ for cofilin, 5′- AGCAGCGGCCTGGTGCCGCGCGGCAGCCAT-3′ and 5′- GGCCGAATTCTTACTTGTACAGCTCGTCCATGCC-3′ for mCherry. The full fusion sequence was amplified by employing the forward primer of cofilin and the reverse primer of mCherry and using both obtained fragments as a PCR template. It was inserted into the vector as a BamHI-EcoRI fragment. Since CAP1 was split in an N-terminal and a C-terminal fragment in two individual vectors, full-length CAP1 was cloned by using the primers 5′-CCCGGAATTCATGGCTGACATGCAAAATCTTGTA-3′ and 5′- AGATGGCAATCCACTCAGTTCTTTTGCCAC-3′ for the N-terminal fragment, and 5′- CTGAGTGGATTGCCATCTGGACCCTCTGTG-3′ and 5′-GAGA CTCGAGTTATCCAGCGATTTCTGTCACTGT-3′ for the C-terminal fragment. Both fragments were fused as described previously by PCR and inserted into a pET-28b(+) vector as an EcoRI-XhoI fragment.

### Bead assays

Previous to functionalization, an aliquot of cOmplete Ni-NTA resin (Roche) was washed three times with water in a clean disposable column. Washed beads were stored in water for up to one week at 4 °C. For functionalization, beads were carefully mixed with a total amount of 10 nmol of either formin, VCA or an equimolar mixture of both per mL of bedded resin. The binding reaction was carried out for 10 minutes on ice and stopped by washing with a 100× excess of water. Protein mixtures for network polymerization experiments were produced by mixing 12 µM profilin, 100 nM capping protein, varying concentrations of cofilin and 1 µM of CAP1 in polymerization buffer (10 mM imidazole, 3 mM MgCl_2_, 0,2 mM CaCl_2_, 1 mM DTT, 1 mM ATP, pH 7.2). The polymerization buffer was designed for slow spontaneous polymerization kinetics, therefore no additional salt was added. For experiments with VCA functionalized beads, the mixture was further supplemented with 300 nM Arp2/3 complex and the concentration of capping protein was reduced by a factor of 10 to generate clearly detectable Arp2/3 clusters. The reactions were started by first supplying the mixtures with 0,5% v/v bedded resin of functionalized beads, immediately followed by addition of 5 µM G-actin with a fraction of 10% labeled monomers.

### Imaging and data acquisition

Confocal images were taken with a 63× oil immersion objective with a numerical aperture (NA) of 1.4 or 20x oil immersion objective with a NA of 0.7 on a Leica TSC SP5. The pinhole was set to 2. The argon laser was set to 50% power and images of 1024 × 1024 pixels were acquired at a scanning speed of 100 Hz with 25% power of the 488-laser line for Atto488-actin and 35% laser power of the 543 helium-neon laser line for mCherry-cofilin. In all experiments, image acquisition was started 1 minute after initiation of the polymerization reaction. Confocal images were used to measure the length of actin networks by use of the Fiji distribution of ImageJ^49,50^. The radial intensity profiles of networks were analyzed employing a python script that identifies the center of beads and averages the intensities from the center towards the outside solution over all slices of the network.

### FRAP assays

FRAP experiments were conducted by using a 63x oil immersion objective with a NA of 1.4 on a Leica TSC SP5 microscope and the Leica FRAP Wizard software. The pinhole was set to 2 and the argon laser was set to 50% power. Images of 512 ×512 pixels were acquired at a scanning speed of 400 Hz with 25% power of the 488-laser line. Bleaching was achieved by use of the “Zoom In” method and defining rectangular regions of interest directly at the bead surface and by setting all laser lines of the 488 lasers to 100%. Analysis of the images were done with the FRAP wizard software by subtracting the background and fitting the photo recovery with a single exponential.

## Supplementary information

Supplementary Information.

Supplementary Information2.

Supplementary Information3.

Supplementary Information4.

## Data Availability

The data that support the findings of this study is available from A.R.B. upon request.

## Code availability

The python code for analyzing radial intensity profiles is available from A.R.B. upon request.

## References

[1] Theriot, J. A. & Mitchison, T. J. Actin microfilament dynamics in locomoting cells. *Nature*., 10.1038/352126a0 (1991). 10.1038/352126a0 2067574

[2] Pollard, T. D., Blanchoin, L. & Mullins, R. D. Molecular Mechanisms Controlling Actin Filament Dynamics in Nonmuscle Cells. *Annu. Rev. Biophys. Biomol. Struct*., 10.1146/annurev.biophys.29.1.545 (2002). 10.1146/annurev.biophys.29.1.545 10940259

[3] Pollard, T. D. Rate constants for the reactions of ATP- and ADP-actin with the ends of actin filaments. *J. Cell Biol*., 10.1083/jcb.103.6.2747 (1986). 10.1083/jcb.103.6.2747 PMC2114620 3793756

[4] Pollard, T. D. & Borisy, G. G. Cellular motility driven by assembly and disassembly of actin filaments. *Cell* (2003). 10.1016/s0092-8674(03)00120-x 12600310

[5] Gressin, L., Guillotin, A., Guérin, C., Blanchoin, L. & Michelot, A. Architecture dependence of actin filament network disassembly. *Curr. Biol*., 10.1016/j.cub.2015.04.011 (2015). 10.1016/j.cub.2015.04.011 25913406

[6] Keren, K., Yam, P. T., Kinkhabwala, A., Mogilner, A. & Theriot, J. A. Intracellular fluid flow in rapidly moving cells. *Nat. Cell Biol*., 10.1038/ncb1965 (2009). 10.1038/ncb1965 PMC2867054 19767741

[7] Michelot, A. *et al*. Actin-Filament Stochastic Dynamics Mediated by ADF/Cofilin. *Curr. Biol*., 10.1016/j.cub.2007.04.037 (2007). 10.1016/j.cub.2007.04.037 17493813

[8] de la Cruz, E. M. How cofilin severs an actin filament. *Biophysical Reviews*, 10.1007/s12551-009-0008-5 (2009). 10.1007/s12551-009-0008-5 PMC2917815 20700473

[9] Suarez, C. *et al*. Cofilin tunes the nucleotide state of actin filaments and severs at bare and decorated segment boundaries. *Curr. Biol*., 10.1016/j.cub.2011.03.064 (2011). 10.1016/j.cub.2011.03.064 PMC3100394 21530260

[10] Shekhar S, Carlier M-F (2017). Enhanced Depolymerization of Actin Filaments by ADF/Cofilin and Monomer Funneling by Capping Protein Cooperate to Accelerate Barbed-End Growth. Curr. Biol..

[11] Wioland, H. *et al*. ADF/Cofilin Accelerates Actin Dynamics by Severing Filaments and Promoting Their Depolymerization at Both Ends. *Curr. Biol*., 10.1016/j.cub.2017.05.048 (2017). 10.1016/j.cub.2017.05.048 PMC5505867 28625781

[12] Carlier, M. F. *et al*. Actin depolymerizing factor (ADF/cofilin) enhances the rate of filament turnover: Implication in actin-based motility. *J. Cell Biol*., 10.1083/jcb.136.6.1307 (1997). 10.1083/jcb.136.6.1307 PMC2132522 9087445

[13] Bertling, E., Quintero-Monzon, O., Mattila, P. K., Goode, B. L. & Lappalainen, P. Mechanism and biological role of profilin-Srv2/CAP interaction. *J. Cell Sci*., 10.1242/jcs.000158 (2007). 10.1242/jcs.000158 17376963

[14] Moriyama, K. & Yahara, I. Human CAP1 is a key factor in the recycling of cofilin and actin for rapid actin turnover. *J. Cell Sci*. (2002). 10.1242/jcs.115.8.1591 11950878

[15] Blanchoin, L. & Pollard, T. D. Interaction of actin monomers with Acanthamoeba actophorin (ADF/cofilin) and profilin. *J. Biol. Chem*., 10.1074/jbc.273.39.25106 (1998). 10.1074/jbc.273.39.25106 9737968

[16] Selden, L. A., Kinosian, H. J., Estes, J. E. & Gershman, L. C. Impact of profilin on actin-bound nucleotide exchange and actin polymerization dynamics. *Biochemistry*, 10.1021/bi981543c (1999). 10.1021/bi981543c 10052948

[17] Wolven, A. K., Belmont, L. D., Mahoney, N. M., Almo, S. C. & Drubin, D. G. *In vivo* importance of actin nucleotide exchange catalyzed by profilin. *J. Cell. Biol*., 10.1083/jcb.150.4.895 (2000). 10.1083/jcb.150.4.895 PMC2175289 10953013

[18] Jansen, S., Collins, A., Golden, L., Sokolova, O. & Goode, B. L. Structure and mechanism of mouse cyclase-associated protein (CAP1) in regulating actin dynamics. *J. Biol. Chem*., 10.1074/jbc.M114.601765 (2014). 10.1074/jbc.M114.601765 PMC4215250 25228691

[19] Jégou, A., Carlier, M. F. & Romet-Lemonne, G. Formin mDia1 senses and generates mechanical forces on actin filaments. *Nat. Commun*., 10.1038/ncomms2888 (2013). 10.1038/ncomms2888 23695677

[20] Bombardier, J. P. *et al*. Single-molecule visualization of a formin-capping protein ‘decision complex’ at the actin filament barbed end. *Nat. Commun*., 10.1038/ncomms9707 (2015). 10.1038/ncomms9707 PMC4660045 26566078

[21] Jansen, S. *et al*. Single-molecule imaging of a three-component ordered actin disassembly mechanism. *Nat. Commun*., 10.1038/ncomms8202 (2015). 10.1038/ncomms8202 PMC4443854 25995115

[22] Cáceres, R., Abou-Ghali, M. & Plastino, J. Reconstituting the actin cytoskeleton at or near surfaces *in vitro*. *Biochimica et Biophysica Acta - Molecular Cell Research*., 10.1016/j.bbamcr.2015.07.021 (2015). 10.1016/j.bbamcr.2015.07.021 26235437

[23] Boujemaa-Paterski, R. *et al*. Network heterogeneity regulates steering in actin-based motility. *Nat. Commun*., 10.1038/s41467-017-00455-1 (2017). 10.1038/s41467-017-00455-1 PMC5608943 28935896

[24] Carlier, M. F. & Shekhar, S. Global treadmilling coordinates actin turnover and controls the size of actin networks. *Nature Reviews Molecular Cell Biology*., 10.1038/nrm.2016.172 (2017). 10.1038/nrm.2016.172 28248322

[25] Antkowiak A (2019). Sizes of actin networks sharing a common environment are determined by the relative rates of assembly. PLOS Biol..

[26] Plastino, J. & Blanchoin, L. Dynamic stability of the actin ecosystem. *J. Cell Sci*., 10.1242/jcs.219832 (2018). 10.1242/jcs.219832 30104258

[27] Machesky, L. M. *et al*. Scar, a WASp-related protein, activates nucleation of actin filaments by the Arp2/3 complex. *Proc. Natl. Acad. Sci*., 10.1073/pnas.96.7.3739 (1999). 10.1073/pnas.96.7.3739 PMC22364 10097107

[28] Blanchoin, L., Pollard, T. D. & Mullins, R. D. R. D. Interactions of ADF/cofilin, Arp2/3 complex, capping protein and profilin in remodeling of branched actin filament networks. *Curr. Biol*., 10.1016/S0960-9822(00)00749-1 (2000). 10.1016/s0960-9822(00)00749-1 11069108

[29] Kovar, D. R., Harris, E. S., Mahaffy, R., Higgs, H. N. & Pollard, T. D. Control of the assembly of ATP- and ADP-actin by formins and profilin. *Cell*, 10.1016/j.cell.2005.11.038 (2006). 10.1016/j.cell.2005.11.038 16439214

[30] Li, F. & Higgs, H. N. The mouse formin mDia1 is a potent actin nucleation factor regulated by autoinhibition. *Curr. Biol*., 10.1016/S0960-9822(03)00540-2 (2003). 10.1016/s0960-9822(03)00540-2 12906795

[31] Blanchoin, L., Boujemaa-Paterski, R., Sykes, C. & Plastino, J. Actin Dynamics, Architecture, and Mechanics in Cell Motility. *Physiol. Rev*., 10.1152/physrev.00018.2013 (2014). 10.1152/physrev.00018.2013 24382887

[32] Andrianantoandro, E. & Pollard, T. D. Mechanism of Actin Filament Turnover by Severing and Nucleation at Different Concentrations of ADF/Cofilin. *Mol. Cell*, 10.1016/j.molcel.2006.08.006 (2006). 10.1016/j.molcel.2006.08.006 17018289

[33] Ressad, F. *et al*. Kinetic Analysis of the Interaction of Actin-depolymerizing Factor (ADF)/Cofilin with G- and F-Actins. *J. Biol. Chem*., 10.1074/jbc.273.33.20894 (2002). 10.1074/jbc.273.33.20894 9694836

[34] Breitsprecher, D. *et al*. Cofilin cooperates with fascin to disassemble filopodial actin filaments. *J. Cell Sci*., 10.1242/jcs.086934 (2011). 10.1242/jcs.086934 PMC4074248 21940796

[35] Carlier, M. F. Actin polymerization and ATP hydrolysis. *Adv. Biophys*., 10.1016/0065-227X(90)90007-G (1990). 10.1016/0065-227x(90)90007-g 2082729

[36] Kotila T (2019). Mechanism of synergistic actin filament pointed end depolymerization by cyclase-associated protein and cofilin. Nat. Commun..

[37] Shekhar, S., Chung, J., Kondev, J., Gelles, J. & Goode, B. L. Synergy between Cyclase-associated protein and Cofilin accelerates actin filament depolymerization by two orders of magnitude. *Nat. Commun*., 10.1038/s41467-019-13268-1 (2019). 10.1038/s41467-019-13268-1 PMC6876572 31757952

[38] Huehn, A. *et al*. The actin filament twist changes abruptly at boundaries between bare and cofilin-decorated segments. *J. Biol. Chem*., 10.1074/jbc.AC118.001843 (2018). 10.1074/jbc.AC118.001843 PMC5900768 29463680

[39] Chan, C., Beltzner, C. C. & Pollard, T. D. Cofilin Dissociates Arp2/3 Complex and Branches from Actin Filaments. *Curr. Biol*., 10.1016/j.cub.2009.02.060 (2009). 10.1016/j.cub.2009.02.060 PMC3711486 19362000

[40] Shekhar, S. & Carlier, M.-F. Single-filament kinetic studies provide novel insights into regulation of actin-based motility. *Mol. Biol. Cell*., 10.1091/mbc.e15-06-0352 (2015). 10.1091/mbc.E15-06-0352 PMC4694749 26715420

[41] Manhart, A. *et al*. Quantitative regulation of the dynamic steady state of actin networks. *Elife*., 10.7554/eLife.42413 (2019). 10.7554/eLife.42413 PMC6417862 30869077

[42] Mizuno, H., Tanaka, K., Yamashiro, S., Narita, A. & Watanabe, N. Helical rotation of the diaphanous-related formin mDia1 generates actin filaments resistant to cofilin. *Proc. Natl. Acad. Sci. USA*, 10.1073/pnas.1803415115 (2018). 10.1073/pnas.1803415115 PMC5984536 29760064

[43] Wioland, H., Jegou, A. & Romet-Lemonne, G. Torsional stress generated by ADF/cofilin on cross-linked actin filaments boosts their severing. *Proc. Natl. Acad. Sci. USA*, 10.1073/pnas.1812053116 (2019). 10.1073/pnas.1812053116 PMC6377502 30692249

[44] Burke, T. A. *et al*. Homeostatic actin cytoskeleton networks are regulated by assembly factor competition for monomers. *Curr. Biol*., 10.1016/j.cub.2014.01.072 (2014). 10.1016/j.cub.2014.01.072 PMC3979332 24560576

[45] Suarez, C. & Kovar, D. R. Internetwork competition for monomers governs actin cytoskeleton organization. *Nature Reviews Molecular Cell Biology*., 10.1038/nrm.2016.106 (2016). 10.1038/nrm.2016.106 PMC5125073 27625321

[46] Spudich, J. A. & Watt, S. The regulation of rabbit skeletal muscle contraction. I. Biochemical studies of the interaction of the tropomyosin-troponin complex with actin and the proteolytic fragments of myosin. *J. Biol. Chem*. (1971). 4254541

[47] Studier, F. W. Protein production by auto-induction in high density shaking cultures. *Protein Expr. Purif*. (2005). 10.1016/j.pep.2005.01.016 15915565

[48] Dürre, K. *et al*. Capping protein-controlled actin polymerization shapes lipid membranes. *Nat. Commun*., 10.1038/s41467-018-03918-1 (2018). 10.1038/s41467-018-03918-1 PMC5915599 29691404

[49] Rueden, C. T. *et al*. ImageJ2: ImageJ for the next generation of scientific image data. *BMC Bioinformatics*., 10.1186/s12859-017-1934-z (2017). 10.1186/s12859-017-1934-z PMC5708080 29187165

[50] Schindelin, J. *et al*. Fiji: An open-source platform for biological-image analysis. *Nature Methods*., 10.1038/nmeth.2019 (2012). 10.1038/nmeth.2019 PMC3855844 22743772

