# Supplementary material for: The dynamics of actin network turnover is self-organized by a growth-depletion feedback
Source: Sci Rep. 2020 Apr 10;10:6215. doi: 10.1038/s41598-020-62942-8 (PMC7148320; doi:10.1038/s41598-020-62942-8)
Supplement: Supplementary file 1 — Supplementary Information. [file 41598_2020_62942_MOESM1_ESM.pdf]

Supplementary Information

The dynamics of actin network turnover is self-organized by a growth-depletion feedback

P. Bleicher (p.bleicher@tum.de), A. Sciortino (alfredo.sciortino@tum.de) and A. R. Bausch* (abausch@mytum.de)

Lehrstuhl für Biophysik E27, Physik-Department, Technische Universität München, Garching, Germany

Movies

Supplementary Movie S1. Formin functionalized beads experience a high actin (magenta) turnover at the bead surface, while cofilin (green) decorates and processes the pointed ends. Undecorated barbed ends at the bead surface only appear in the presence of ATP through constant elongation of ATP-bound monomers at the bead surface. Due to the network polymerization, fluctuations of the bead in z-direction can be observed. The depicted time is in minutes.

Supplementary Movie S2. At ideal concentrations of cofilin, spinning and translational movements of the beads can be observed. The spinning occurs over an extended time frame that outlives a single polymerization cycle. Shown is only the cofilin fluorescence channel, the depicted time is in minutes.

Supplementary Movie S3. When Arp2/3 and formin networks are colocalized at the bead surface, a dense cortex directly at the bead surface can be polymerized by VCA/Arp2/3 even in the presence of 2 µM cofilin. Only when the concentration is increased from 2 to 3 µM cofilin, the dense network gets disassembled. The depicted time is in minutes.

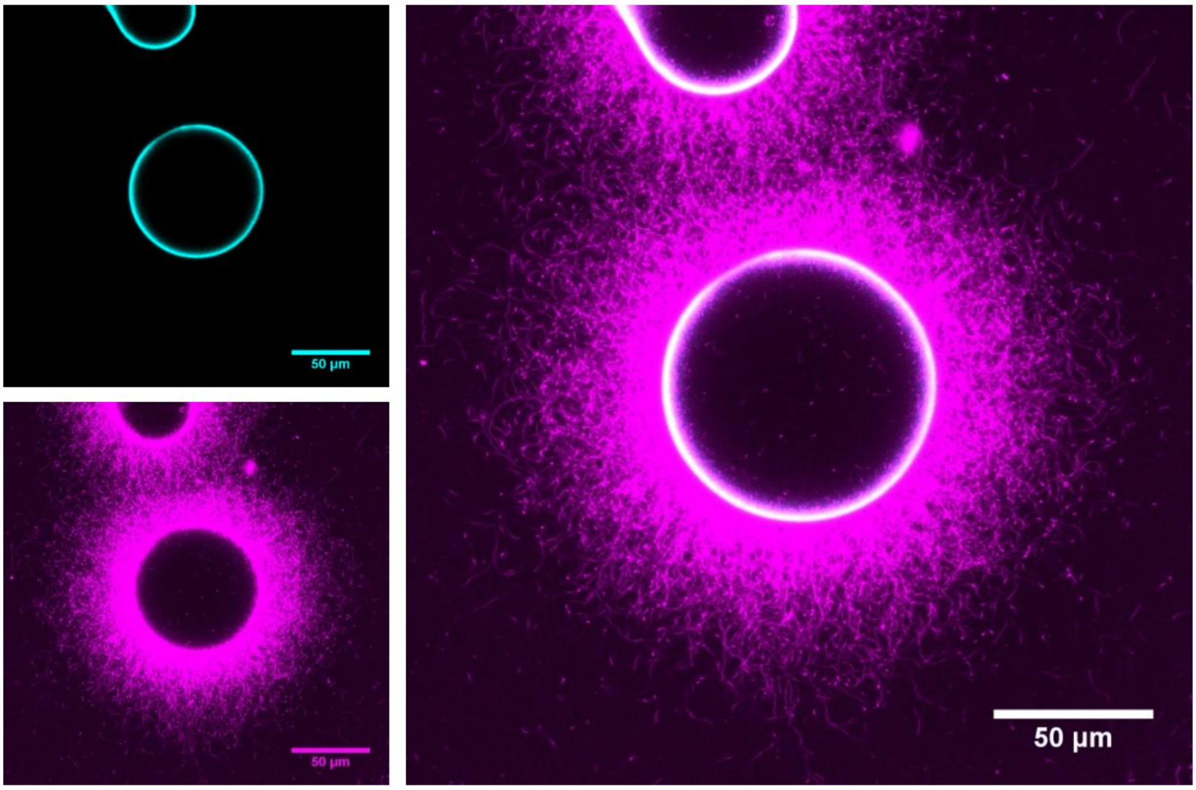

Supplementary Fig. S1 Resolution of individual filaments with phalloidin staining and localization of GFP-formin

For visualization GFP-formin (cyan, top left) is used to confirm the localization at the bead surface. By means of confocal microscopy it can be confirmed that the formin is exclusively bound to the surface and that washing steps with buffer remove unbound formin adequately. In this experiment no cofilin, but Alexa Fluor 647 Phalloidin (Thermo Fisher Scientific) is used to label actin (magenta, bottom left). Since Phalloidin only binds to filaments, monomers are not visible, in contrast to all other presented experiments where covalently labeled actin monomers are used. A higher contrast can be achieved with phalloidin, however colocalization of phalloidin and cofilin was not feasible as phalloidin interferes with filament dynamics and cofilin binding. The network is now clearly visualized and individual, unbundled filaments can be observed emerging from the functionalized bead surface.

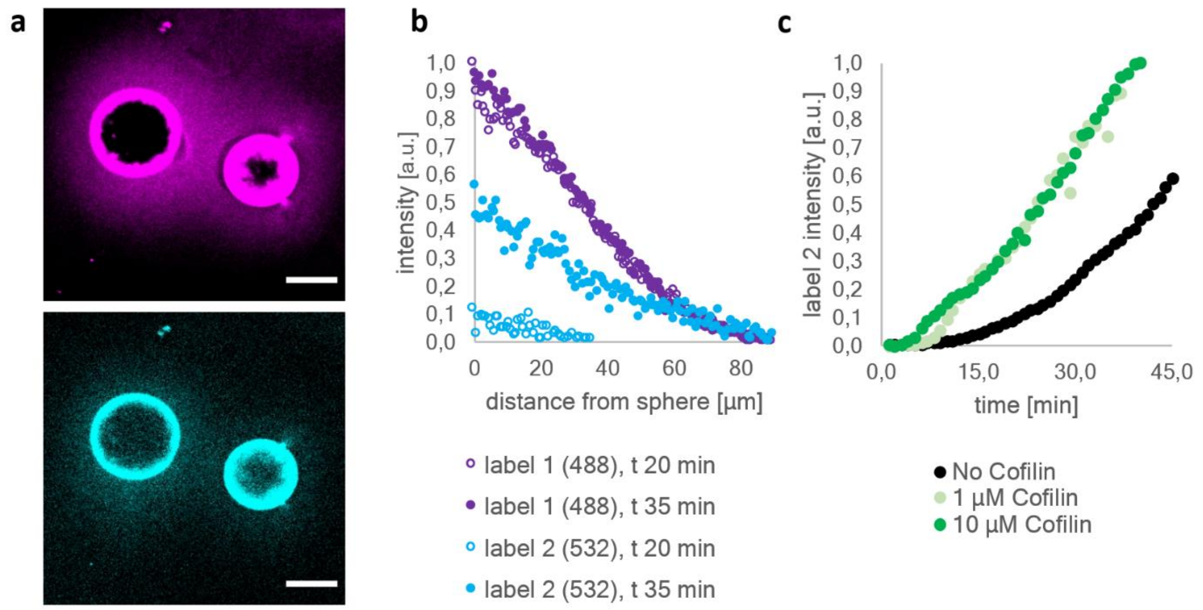

Supplementary Fig. S2 Introduction of monomers labeled with a different fluorescent color to networks polymerized on formin functionalized agarose beads.

Formin functionalized agarose spheres were incubated in a polymerization mixture containing 12 µM profilin, 100 nM capping protein and 5 µM actin with 10% monomers fluorescently labeled with an Atto 488 NHS-ester. After 30 minutes of initial incubation, an additional amount of 500 nM of monomers labeled with an Atto 532 NHS-ester were added. (**a**) Incorporation of the second label (cyan, bottom) occurs from the bead surface. The initially polymerized network containing the first label (magenta, top) remains mostly unchanged. No unlabeled areas appear in the initially polymerized network where the 532-monomers are incorporated, indicating that monomers with both labels are still available and built into the network together. (**b**) Intensity profiles of the networks for both monomer types, at 20 minutes and 35 minutes after addition of the 532-monomers. At 20 minutes the incorporation of the second monomers into the network can be observed. The lag time of 20 minutes can be explained with the slow diffusion of the monomers through the optical chamber, as mixing would disturb the initially polymerized networks. 35 minutes after addition of the second monomers, more monomers have been built into the network. The growth occurs specifically from the surface towards the outside solution, revealing that the filaments are indeed most likely oriented with the barbed ends bound at the bead surface and the pointed ends extend towards the outside solution. The intensity values have been normalized by their respective maximal value at the bead surface. Here, formins get saturated with actin and therefore have an intensity value that depends only on the density of formins. Additionally, the background intensity of the monomers has been subtracted. (**c**) The

kinetics of the incorporation of the second monomers is faster in the presence of cofilin and 1 µM CAP1. For both cofilin concentrations, 1 µM and 10 µM, the incorporation of the second monomer is faster, although no difference in incorporation speed can be observed between 1 µM and 10 µM cofilin. This is in agreement with further analysis of the internal dynamics of this system (Fig. 3). An offset time accounting for the lag time due to diffusion of the additional monomers has been subtracted and the intensity values have been normalized as described previously.

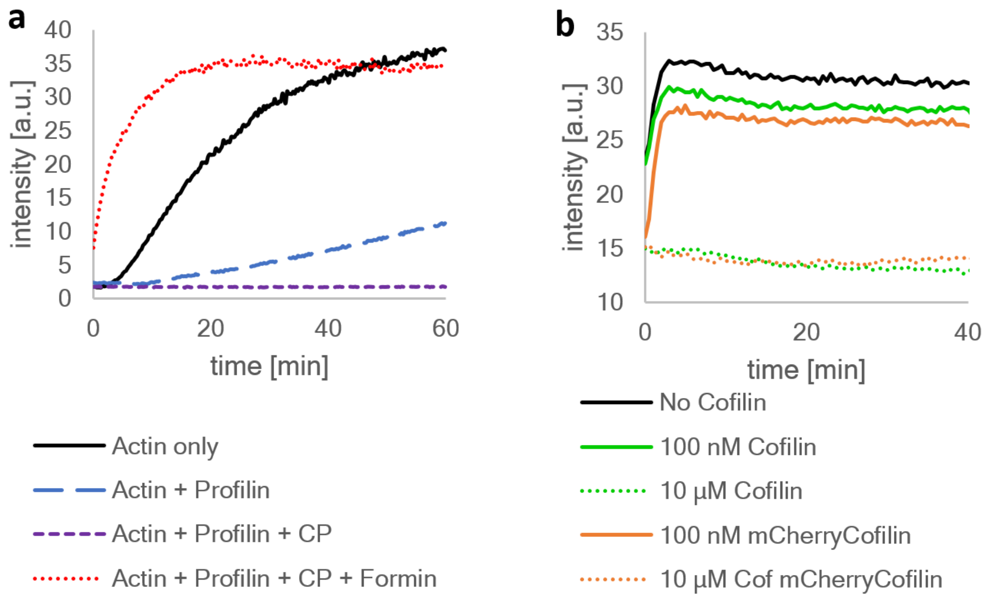

Supplementary Fig. S3 Confirmation of the functionality of actin, cofilin, profilin, capping protein and formin and the activity of the fluorescent fusion protein mCherry-cofilin compared to native human cofilin.

The functionality of all proteins is confirmed by means of pyrene assays that monitor the polymerization of actin via the fluorescence of a 20% fraction of pyrene actin. This is relevant as relatively low salt concentrations are used in the polymerization buffer (10 mM imidazole, 3 mM $MgCl_2$, 0,2 mM $CaCl_2$, 1 mM DTT, 1 mM ATP, pH 7.2). Actin polymerization in these conditions is possible, but expected to be slower compared to polymerization in the presence of KCl. (**a**) Pyrene-actin polymerization in polymerization buffer with 3 µM actin only (black line), 3 µM actin and 12 µM profilin (blue dashed line), 3 µM actin, 12 µM profilin and 120 nM capping protein (magenta dashed line), 3 µM actin, 12 µM profilin, 120 nM capping protein and 1 µM formin (red dotted line). While actin polymerizes normally alone, profilin and capping protein together suppress the polymerization completely. In the presence of formin the

polymerization is significantly faster and a plateau is reached already after 10 minutes, even in the presence of profilin and capping protein. (**b**) The question arises if the activity of the mCherry-cofilin construct is altered compared to native cofilin. To this end, we vary the cofilin concentration in an actin polymerization mixture with 12 µM profilin, 100 nM capping protein, 1 µM CAP1, 300 nM VCA and 300 nM Arp2/3 and 3 µM actin with a 20% fraction of pyrene labeled actin. At 100 nM (green and orange solid line) cofilin, the polymerization is essentially equal to the mixture without cofilin (black line). Although the network is debranched under the same conditions on agarose spheres, the branches display almost the same pyrene fluorescence as the crosslinked network without cofilin. However, at 10 µM cofilin (green and orange dashed lines), no polymerization can be observed. This is most likely due to the depolymerizing effect of CAP1 on cofilin saturate branches. Due to complete disassembly, no branches form. These effects are the same for both mCherry-cofilin and native cofilin, which shows that the disassembly activity of the fusion cofilin protein is not altered. The data point at 0 minutes represents the start of the measurement which was 45 s after the initiation of the polymerization reaction.

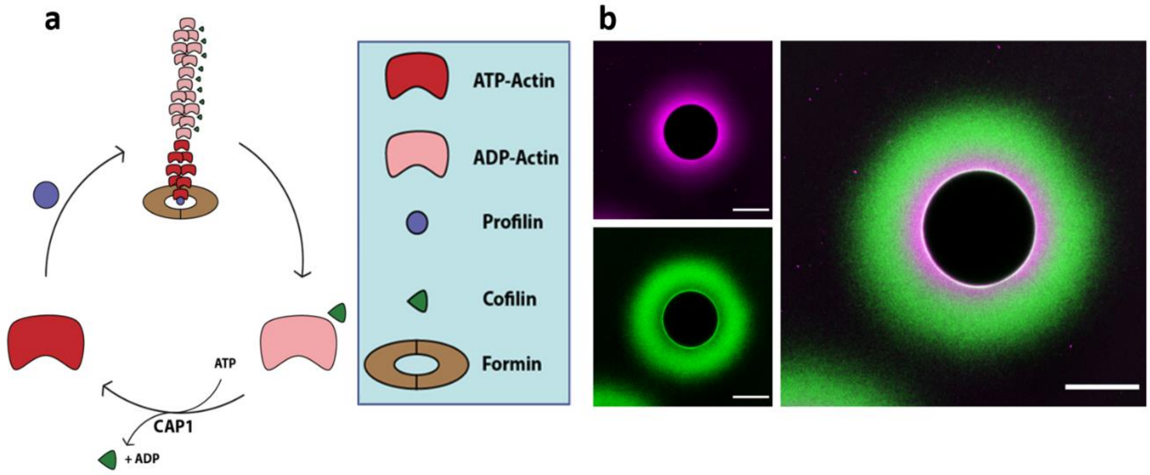

Supplementary Fig. S4 Reconstitution of a dynamic actin network sustained by formin and cofilin.

(**a**) Protein components involved in the formation of a self-replenishing network: Actin (labeled 10% with Atto 488 NHS ester, red), His-tagged formin (brown,) profilin (blue), mCherry-cofilin (green) and CAP1. The recycling cycle can be divided into three steps: Elongation of the growing chain with profilin bound actin monomers at the barbed end, disassembly facilitated by cofilin at the aged fraction of the filament, nucleotide

exchange of ADP with ATP on cofilin bound monomers catalyzed by CAP1. (**b**) His-tagged formin was immobilized on NiNTA-coated agarose beads with an average diameter of 60 µm. Actin polymerization (magenta) is restricted to the surface of the bead, while cofilin (green) decorates from the pointed ends inwards on the filaments that grow normal to the surface. Scale bars are 50 µm.

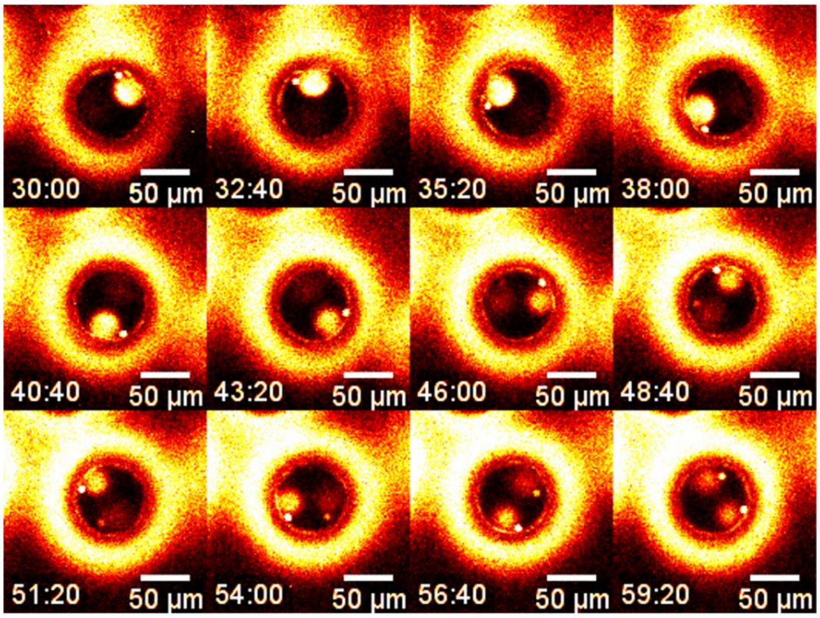

Supplementary Fig. S5 Spinning of beads as a consequence of actin turnover at the bead surface

In the presence of ideal concentrations of cofilin, spinning of beads can be observed. Here, a network is formed by formin functionalized beads in the presence of 1.5 µM mCherry-cofilin. Depicted are individual frames of a timeseries, after 30 minutes of initial polymerization at a framerate of 80 s. For better visualization, only the fluorescence of the cofilin channel is shown.

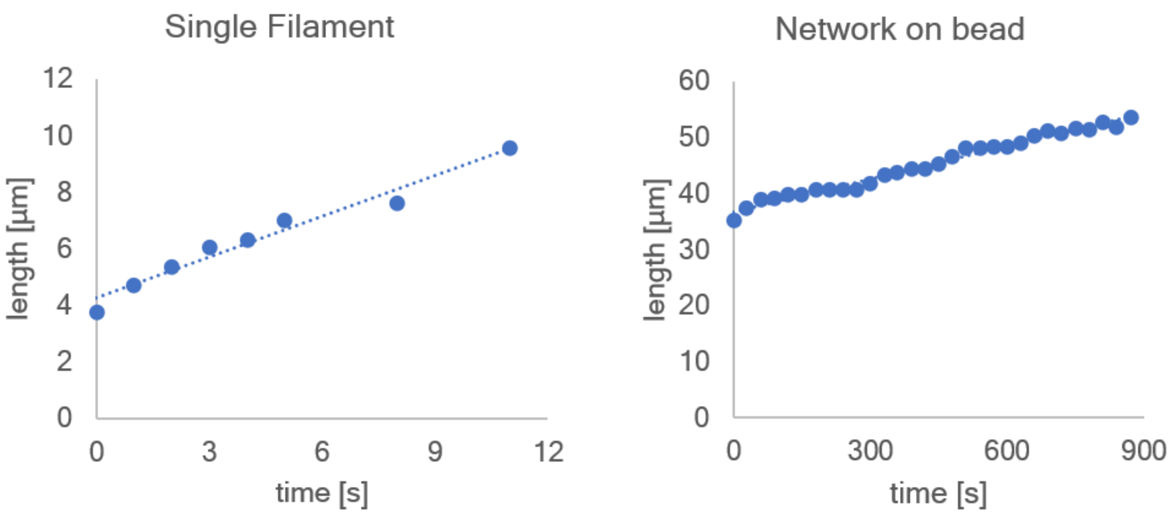

$$v_o = k_0\, \delta\, G \qquad \delta = 3 \text{ nm}$$

Elongation speed of single filament (TIRF):

$$v_o \approx 0.2\ \frac{\mu m}{s} \qquad k_o = 13.3\ \mu M^{-1} s^{-1}$$

Elongation speed on bead:

$$v_o \approx 0.02\ \frac{\mu m}{s}$$

Local monomer depletion:

$$G_1 \approx \frac{1}{10} G_0$$

Supplementary Fig. S6 Elongation speed of single filaments in TIRF microscopy and network elongation speed on beads

Single filaments are polymerized by immobilizing a formin antibody (Anti-DIAPH1, abcam) diluted 1:1000 on a cleaned microscope slide. After incubation with formin, a protein mix equivalent to the mix used for bead measurements is added to the in this way functionalized microscope slide. Here, the polymerization of filaments is monitored by means of TIRF microscopy.

For bead networks, the network width is measured after an initial polymerization of 10 minutes. Assuming the filaments elongate normal to the bead, the network width can be used to account for the growth rate of individual filaments. For a single filament, the rate k_0 is close to 10 $\mu M^{-1} s^{-1}$ and the elongation speed 0.2 µm/s, ten times faster than

the elongation speed on a bead, which is 0.02 µm/s. The elongation speed depends linearly on the monomer concentration, thus the initial global monomer concentration G_0 drops to a tenth of its original value at the elongation sites. This local concentration is defined as G_1, the local monomer concentration ten minutes after the initiation of the experiment.

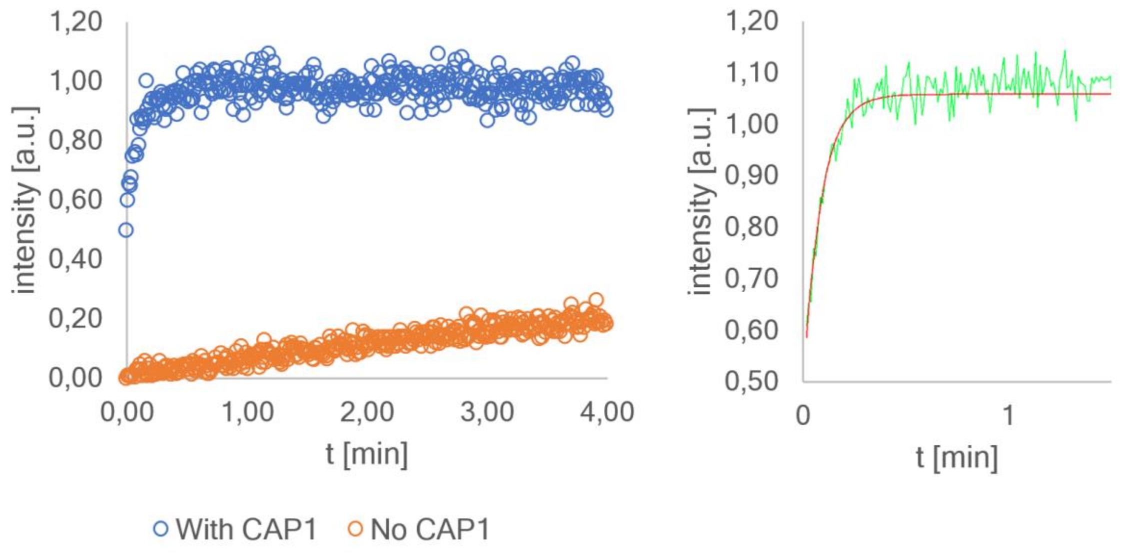

Supplementary Fig. S7 CAP1 dependent monomer turnover determined by FRAP recovery

The FRAP recovery of Atto 488 NHS-ester labeled actin is measured in the presence of 2 µM Cofilin with or without 1 µM CAP1. The recovery rates are extracted with a single exponential fit with the Leica FRAP wizard software. For samples without CAP1, a plateau value cannot be reached. For the shown experiment with 1 µM CAP1, the extracted FRAP recovery rate is 5,1 s. For all other cofilin concentrations, the recovery rate is at least double for samples with CAP1 when compared to samples without CAP1. An example for a single exponential fit achieved with the Leica FRAP wizard software is shown on the right, where the data is shown in green and the corresponding exponential fit as red line.

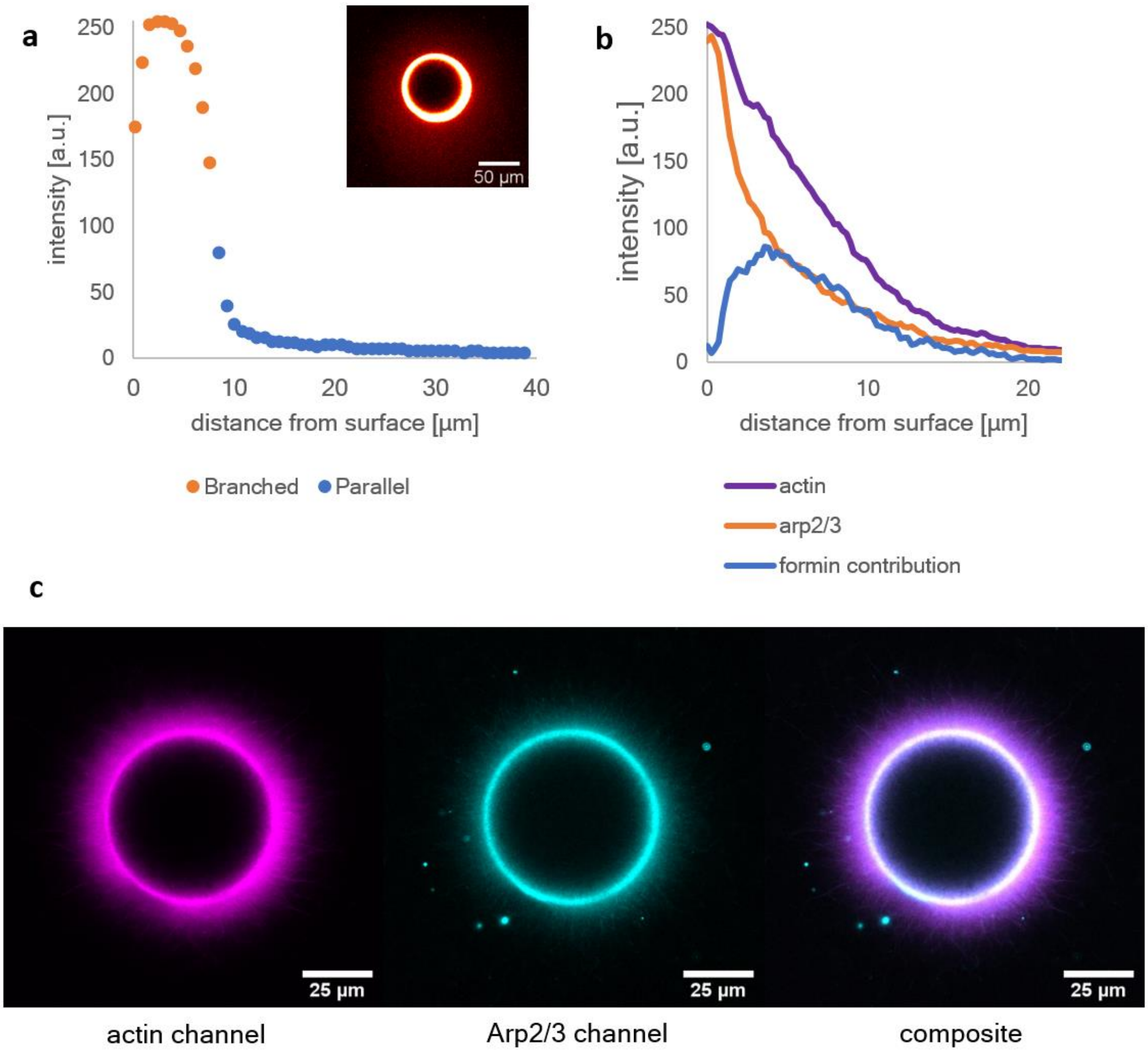

Supplementary Fig. S8 Visualization of the network colocalization in the presence of VCA/Arp2/3-complex and formin

Arp2/3 and formin are colocalized on the same surface. Two different types of networks form in the presence of 5 µM actin, 12 µM profilin 100 nM capping protein and 1.5 µM cofilin. (**a**) By adjusting the lookup table, the colocalized formin network can be visualized (confocal image corresponding to the depicted intensity profile). The intensity profile reveals a difference in intensities which accounts for the different densities of the colocalized networks. An arbitrary threshold (40% of maximal intensity in a.u.) is defined to distinguish between the branched and the formin polymerized part of the network. For geometrical reasons, the profile decreases over time. Therefore, to compensate for the dependence on the distance from the surface, the ratio between the intensities times the corresponding distances is used to calculate the intensity ratio. By calculating the intensity ratio of the branched to the formin polymerized network in that manner, the intensity of the branched part of the network is 6 times higher. (**b**) Intensity line plot corresponding to actin channel and Arp2/3 complex channel depicted

in (**c**). Arp2/3 complex labeled with an Atto647N-maleimid dye is used to visualize the localization of Arp2/3 to confirm that the dense part of the network is indeed branched. The data shows that the Arp2/3 complex is colocalized with the densest area of the actin, which is in close proximity to the surface. This indicates that the 6 times higher intensity of this fraction of the network is indeed due to branching activity. With increasing distance, less Arp2/3 is present compared to the fluorescence of actin. By subtracting the Arp2/3 fluorescence from the actin fluorescence, the contribution of formin induced polymerization to the network can be roughly visualized.

S9 Determination of the nucleator density on beads and the contribution of global actin concentration during turnover experiments

By drying 1 g of resin in a hot oven, we determine the concentration of agarose in the resin. The dry mass m_a of agarose was 0.0991 g. The weight to volume percentage of agarose is then given by

$$^{w}/_{v} = \left(\frac{m_a}{\frac{(1-m_a)}{\rho_w} + \frac{m_a}{\rho_a}} \right) = 0.098 \frac{g}{mL}$$

with the density of water ρ_w and the density of agarose ρ_a. The weight m_b of a single bead can be calculated by multiplying the density of agarose with the volume of an average sphere with a diameter d of 60 µm. We can then calculate the number of beads N_b per mL with the expression

$$N_b = \frac{^{w}/_{v}}{m_b} = \frac{^{w}/_{v}}{\frac{\pi}{6} d^3 \rho_a}$$

Hereby we determine $N_b \approx 10\ 000$ per mL of resin. For all experiments, 10 nmol of protein is used to functionalize one mL of resin. Then 1 pmol protein are present on a single sphere, resulting in a density of 0.05 molecules per Å^2 on the surface.

We measured that the elongation rate k_0 of formin is 13.3 µM^{-1}s^{-1} (Supplementary Fig. S4). Ten minutes after the initiation of the bead experiments, in the absence of at least 2 µM cofilin and CAP1, the elongation slows down by a factor of ten. If we assume a linear slowdown from 5 µM to 0.5 µM monomers, a maximum of 22 000 monomers can be polymerized by a single formin within five minutes. For each bead experiment with a total volume of 100 µL, 0.5 µL of functionalized beads were used, thus 5 pmol formin

are present. If all formins are active and diffusion is not accounted for, a maximum of 110 nM actin can potentially be polymerized into a network, which equals 22 % of the total monomeric actin. Consequently, the slowdown of 90 % elongation speed cannot be explained by a change in global actin concentration and must in fact be a consequence of local monomer depletion at the elongation sites.
